# Review Article: Immune Landscape and Immunotherapy Options in Cervical Carcinoma

**DOI:** 10.3390/cancers14184458

**Published:** 2022-09-14

**Authors:** Kousain Kousar, Tahir Ahmad, Faiza Naseer, Salik Kakar, Sadia Anjum

**Affiliations:** 1 Industrial Biotechnology, Atta ur Rahman School of Applied Biosciences, National University of Sciences and Technology, Islamabad 44000, Pakistan; 2 Shifa College of Pharmaceutical Sciences, Shifa Tameer e Millat University, Islamabad 44000, Pakistan; 3 School of Health Sciences, National University of Sciences and Technology, Islamabad 44000, Pakistan; 4 Department of Biology, University of Hail, Hail 81442, Saudi Arabia

**Keywords:** immunotherapy, cervical carcinoma, monoclonal antibodies, pembrolizumab, HPV, oncolytic virus, adoptive t-cell therapy, immune checkpoint inhibitors, immune evasion

## Abstract

**Simple Summary:**

Cervical cancer is one of the most common cancers with a high mortality rate, especially in women of reproductive age. A lot of treatment modalities are being used in clinical practice but they come with a wide range of toxic side effects, the relapse of cancer, and a low disease-free survival rate. Immunotherapy has revolutionized the treatment landscape of cervical cancer as it focuses majorly on agents that stimulate the body’s own immune system against tumor cells. A deeper understanding of immune system players and immune perturbations in the onset and progression of cervical cancer can pave the way to better treatment with zero relapse. Immunotherapy holds the key to a cancer-free future. This review summarizes the immune players that are perturbed in cervical cancer, and immunotherapy options that are being exploited, alone or in combination, for the treatment of cervical carcinoma in women.

**Abstract:**

Carcinoma of the cervix is one of the most common cancers that claims women’s lives every year. Despite preventive HPV vaccines and conventional cancer treatments, approximately 273,000 women succumb to cervical carcinoma every year. Immune system perturbations help malignant cells in immune evasion, tumor establishment, invasion, and metastasis. An insight into immune system players that promote or suppress cervical cancer is important for the development of more targeted therapies with the fewest side effects. Immunotherapy has emerged as the most compliant approach to target cancer because it utilizes a natural course of action to stimulate the immune system against cancer cells. The major immunotherapy approaches for cervical carcinoma include monoclonal antibodies, immune checkpoint blockade therapy, adoptive cell transfer therapies, and oncolytic viruses. In October 2021 the FDA approved pembrolizumab in combination with chemotherapy or bevacizumab as a first-line treatment for cervical cancer. A recent breakthrough has been made in the cancer immunotherapy regimen in which a monoclonal antibody dostarlimab was able to completely cure all colorectal cancer patients, with disease-free progression after 6 months and counting. This creates hope that immunotherapy may prove to be the final nail in the coffin of this centuries-long prevalent disease of “cancer”.

## 1. Introduction

Every year approximately 500,000 women are diagnosed with invasive cancer of the cervix throughout the world, killing 273,000 women. More than 70% of cancer patients report the very progressive stage of malignancy. In 2020 alone, 604,127 women were diagnosed with cervical cancer worldwide (The International Agency for Research on Cancer (IARC). The current conventional treatment options are surgical tumor resection, radiotherapy, chemotherapy, or a combination of these; these options are not very efficient in the case of advanced tumors since the tumor spreads to the reproductive system, urinary tract, and bone marrow [1]. This review focuses on immune perturbations associated with cervical cancer and different immunotherapy options that can prove to be fruitful in curbing this deadly carcinoma of the cervix.

### 1.1. Risk Factors Associated with the Onset of Cervical Cancer

The most common risk factor associated with the onset of cervical cancer is infection with human papillomavirus (HPV). There are five genera of HPV: α, β, γ, μ, and ν. From these genera, types 16, 18, 31, 33, 35, 39, 45, 51, 52, 56, 58, 59, and 68 have been identified as high risk and types 6, 11, 42, and 44 have been identified as low-risk HPV types. In 90% of cervical cancer cases, HPV types 16 and 18 are found to be involved [2]. In a study conducted worldwide, the prevalence rate of HPV genotypes associated with cervical cancer were HPV16 (61%), HPV18 (10%), HPV31 (4%), and HPV33. Other factors include high parity, smoking, very young age at the time of first coitus, multiple sexual partners, and low socioeconomic status [3].

As compared to other high-risk HPV genotypes, infection due to HPV16 and 18 are likely to lead to invasive cervical cancer in less time, so they are more oncogenic variants of HPV [4]. The onset of precancerous lesions and cervical cancer occurs due to the overexpression of HPV oncogenes E6 and E7 in persistent high-risk HPV infections. The expression of E6 and E7 induces numerical and structural chromosome instability leading to aneuploidy and chromosome missegregation; it also directly interferes with critical cell cycle pathways and genes (pRB, p53,c-myc) leading to the disruption of apoptosis, abnormal cell proliferation, and malignant transformation [5].

### 1.2. The Immunological Landscape of Cervical Cancer

**Figure 1:**
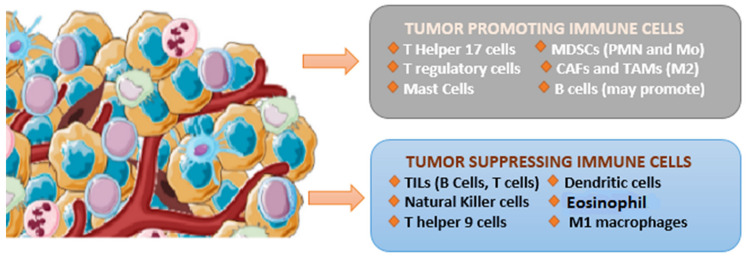
Tumor-suppressing and tumor-promoting cells in the tumor microenvironment of cervical cancer.

The tumor microenvironment (TME) is a major player in the suppression of the antitumor response. Chronic inflammation, immune checkpoint inhibition, and propagation of a vascular regenerative environment topple the immune cells’ population equilibrium in favor of a tumor supportive environment. These abnormal dynamics of the tumor microenvironment play a pivotal role in the initiation, progression, and immune evasion of cancer cells. TME is heavily infiltrated by the abundance of different types of immune cells including tumor-associated fibroblasts, natural killer (NK) cells, macrophages, dendritic cells, T cells, and B cells. These TME-associated cells can be classified into two groups, namely, immune suppressive cells and immune stimulatory cells, depending upon the function they perform as shown in Figure 1 [6] TME is also associated with tumor-infiltrating lymphocytes, which are important players in cervical cancer immune evasion and metastasis.

### 1.3. Tumor-Infiltrating Lymphocyte (TIL)

The interaction between immune cells and TME is critical for immune evasion and cervical tumor initiation. The ambiguous role of TILs at the tumor site is considered to be the initiation factor behind cervical cancer onset. An antitumor cytotoxic cellular response is marked by antigen-presenting cells, CD4 and CD8, and other lymphoid elements. CD4+T cells, also called helper T lymphocytes, function to activate CD8+T cells, also called cytotoxic T cells. Based on the stimulatory function they perform, CD4+ cells are categorized into four major subgroups: regulatory T cells, Th1, Th2, and Th17 [7]. These subsets of T cells exert their positive and negative role by secreting different cytokines in order to maintain the normal immune function. Interferon-γ (IFN-γ) and IL-12 are two important cytokines secreted by Th1 cells. IL-12 is a major cytokine involved in the induction and maintenance of the Th1 cell population, the maintenance of IFN-γ responses, and IL-10, which modulates the cell-mediated immune response by promoting Th2 cells. Th17 is found to secrete a pro-inflammatory cytokine IL-17, which is involved in proliferation, invasion, and angiogenesis in cervical cancer [8]. Both Treg cells and Th17 cells are derived from common precursor naïve CD4 T cells, and both need TGF-β for initial differentiation, and after differentiation both perform opposite functions. Treg cells suppress inflammation and autoimmunity, maintain immune homeostasis, and promote self-tolerance, but at the same time, they suppress the immune system from targeting tumor cells, whereas Th17 cells support inflammation and autoimmunity but also promote a tumor supportive environment. Specifically in cervical cancer, Th17 promotes carcinogenesis by the effect of microRNAs miR155 and miR146-a [9]. Newly discovered T helper 9 (Th9) cells are found to play a crucial role in suppressing malignant transformation and curbing the progression of cervical cancer through its signatory cytokines IL-9 and IL-21 by enhancing apoptosis, suppressing proliferation, and stimulating the expression of e-cadherin (controlling extravasation) and MHC-I (increasing cytotoxic T cell response) on Hela cells. This suggests their antitumor effect on cervical cancer [10]. CD8+ cytotoxic T cells are the most preferred cells to eradicate cancer cells, but these CTLs become dysfunctional and inadequate due to immunosuppression and immune tolerance toward cancer cells. Studies have suggested that chemotherapeutic drugs such as cisplatin and immunotherapy approaches lead to the increased infiltration of CD8+ cells in the tumor microenvironment [11]. TME-mediated modulation of tumor-infiltrated dendritic cells suppresses their ability to prime a potent cytotoxic immune response by CD8+T cells. Therefore, during cancer progression, CTLs encounter dysfunction and exhaustion due to immune-related tolerance and immunosuppression within the tumor microenvironment (TME), all of which favor adaptive immune resistance favoring cervical neoplasia [12].

### 1.4. Tumor-Associated Macrophages (TAMs)

Other important immune-associated cellular components that play a pivotal role in changing the dynamics of the tumor microenvironment in favor of carcinogenesis are tumor-associated macrophages. The function of TAMs includes the growth of tumors, metastasis, drug resistance, and invasion [13]. Macrophages can be divided into two categories, namely, classical M1 macrophages and alternative M2 macrophages. M1 or classical macrophages are responsible for identifying cancer cells from normal cells and then eradicating tumor cells. M1 macrophages apply two mechanisms for the effective killing of tumor cells: (i) through directly mediating cytotoxicity against killing cancerous cells. For example, by tumor-killing reactive ROS and NO molecules, which are cytotoxic to tumor cells. This is a slow process, involving multiple mechanisms, and generally takes at least 1 to 3 days [14]. (ii) The other mechanism used by M1 macrophages is antibody-dependent cell-mediated cytotoxicity (ADCC) against tumor cells, which requires the involvement of antitumor antibodies and takes less time, usually a few hours, to kill cancer cells [15]. M1 macrophages are activated through granulocyte colony-stimulating factors (GM-CSF) and toll-like receptors in response to lipopolysaccharides (bacterial products) and IFN-γ. M1 macrophages activate the Th1 immune response and function majorly in the killing of pathogens and eradication of tumor cells. They highly express MHC-II molecules and release co-stimulatory molecules such as reactive nitrogen species, reactive oxygen species TNF-α, IL-12, IL-13, and CD86/CD80 [16]. M2 has a weakened ability for antigen presentation and therefore has low antitumor activity. They have an increased ability for tissue remodeling and angiogenesis. They promote angiogenesis by the release of molecules including cyclo-oxygenase-2 and matrix metalloproteases (MMP-12, MMP-9, MMP-7, and MMP-2), which are associated with the progression and regulation of angiogenesis in cervical cancer [17].They promote epithelial to mesenchymal transition (EMT), which is the hallmark of invasion and metastasis, thus promoting cervical cancer. Polarization of tumor-associated macrophages toward the M2 phenotype correlates with a reduced response to chemoradiation therapy and short survival in patients with regionally advanced cervical carcinoma. M2 macrophages increase the expression of IL-10 and CD163 and IL-10. CD163 is a favorable tumor marker for predicting the malignant transformation and metastatic potential of cervical cancer [18].After M1 macrophages encounter squamous cell cervical carcinoma cells, they undergo the transformation from M1 phenotype to M2 phenotype due to the lactate secreted by cervical cancer cells (cancer cells prefer lactate metabolism over glucose metabolism and therefore maintain slightly acidic TME) that promotes the progression of cervical cancer [19].

### 1.5. Cancer-Associated Fibroblasts (CAFs)

Like TAMs, cancer-associated fibroblasts (CAF) are major cells in the stroma of cervical tumors that promote invasion and metastasis. In normal conditions, they are abundantly present in a dormant state in connective tissues; they become transiently active during times of tissue repair and remodeling. They are involved in the modulation of the inflammation, differentiation, and proliferation of endothelial cells and deposition of the extracellular matrix (ECM) [20]. Fibroblast activation signals include transforming growth factor beta (TGF-β) and lysophosphatidic acid that enhance the activity of a transcription factor SMAD, which regulates the expression of alpha-smooth muscle actin (α-SMA). α-SMA provides a highly contractile phenotype to fibroblasts (also called myofibroblast) [21]. This activated fibroblast can now efficiently communicate with epithelial, mesenchyme, and immune cells through the release of cytokines and chemokines. Along with physical and chemical barriers that hamper immune cells from mounting a potent antitumor response at the site of a tumor, immune checkpoint ligands are expressed on TAMs and CAFs [22]. According to a study, CAFs expressing PD-L1 and PD-L2 (ligands to PD-1) were major contributors to immune cells’ energy or immune evasion [23]. According to research, it was found that the transition of stromal fibroblasts into cancer-associated fibroblasts is mediated by Wnt2B, which is found to be enriched in exosomes secreted by cervical cancer cells. These Wnt2B signal transduction proteins interact with the fibroblast to mediate activation of the Wnt/β-catenin signaling pathway, diminishing the concentration of Wnt2B inhibiting these signal transduction pathways, and ultimately leading to a low concentration of CAFs in cervical cancer lesions [24].

### 1.6. Dendritic Cells (DCs)

Antigen-presenting cells are major activators of CD4+ and CD8+T lymphocytes. Though the response is not focused on cervical cancer cells, dendritic cells play a role as potent APCs to present tumor antigens for the activation of the Th1-based CTLs’ response [25]. It has been observed that the concentration of DCs is low, while that of Treg cells is high in cervical cancer lesions, which might be significantly associated with the persistence of hrHPV. Due to this, DCs lose their antigen-presenting ability gradually against tumor cells [26]. It has been proposed that DCs lose their sensitivity to cervical cancer cells due to the secretion of RANKL by cancer cells. RANKL is an apoptosis regulator gene, a ligand for the receptor RANK, and controls cell proliferation. It is a receptor activator of the nuclear factor kappa-B ligand, which is a TNF family member. RANKL along with Treg is a promising candidate for immune evasion in cervical cancer. Dendritic cells are activated by RANKL to release multiple activating cytokines [27].

### 1.7. B Cells

B cells, which are produced in germinal centers, exert an antitumor response by the secretion of antibodies and cytokines. A recent study suggested that B cells tend to have a particular role through an immunosuppressive cytokine IL-10 in the progression of HPV-mediated cervical cancer in a mouse model [8].Another study found an increased concentration of B cells and IL-10 in human cervical cancer samples, suggesting its significant role in cervical cancer progression [28]. Another recent study implicated the role of B cells in improving CC squamous carcinoma, which may be activated by PD-1 blockade and radiation therapy. The analysis of data from over 800 patient samples from cervical cancer and head and neck squamous carcinoma (HNSCC), single cells, and RNA sequencing analysis, revealed that following PD-L1 blockade therapy, a dramatic increase in B cell germinal centers and increased IgM and IgG responses were observed [29]. Still, data confirming the role of B cells in the cervical cancer microenvironment are not sufficient and require more research. More reports are needed to support B cells’ role in CC [30].

### 1.8. NK Cells

Natural killer cells play an important role in immunity against cervical cancer lesions through the secretion of various cytokines. In the tumor microenvironment, IL-2 stimulates NK cells. NK cells have been found to fight against cervical cancer through the secretion of tumor necrosis factor-alpha (TNF-α) and interferon-gamma (IFN-γ) [31]. NK cells are major effectors against cancer cells without antigen presentation stimuli. In other words, NK cells are the primary effectors to recognize abnormal cells without an antigen presentation process. A type 2 lectin-like family of transmembrane proteins, NKG2D, have been found to be associated with HPV-induced cancers and immune surveillance, suggesting the role of the NKG2D gene family in influencing cytotoxicity and the susceptibility of NK cells in cervical cancer [32]. While NKp46, NKG2D, and NKp30 are NK activating receptors, NKG2A CD158a, and CD158b are inhibitory NK receptors [33]. A study suggested that NK cells in cervical cancer are not only associated with prognosis but also improve immune clearance. A clinical trial revealed that NK cell concentration increased and tumor size decreased after four cycles of chemotherapy stages Ⅱb–шb of cervical squamous cell carcinoma [34]

### 1.9. Myeloid-Derived Suppressor Cells (MDSCs)

Myeloid-derived suppressor cells (MDSCs) are narrowly associated with tumor progression, staging, prognosis, and clinical therapeutic efficacy; thus, MDSCs have tumor progression and an immunosuppressive role [35]. In a study conducted on an HPV-mediated CC mouse model, scientists found that through IL-6-JAK-STAT3 (signal transducer and activator of transcription 3) signaling, MDSCs such as PMN (polymorphonuclear cells) and Mo (Monocytes) promote an immunosuppressive activity [36]. Moreover, IL-10 can regress cancer growth by inhibiting IL-6 release while activating STAT3 signaling in cervical carcinoma [37].MDSCs are responsible for the insufficient stimulation of APCs and immune reactions to tumor antigens, the impaired activation of CD8+T cells, and, ultimately, for hampering the therapeutic efficacy of immunotherapy in HPV-mediated cervical cancer [38].

### 1.10. Immunotherapy Approaches for the Treatment of Cervical Cancer

Immunotherapy is a promising approach; a kind of biological therapy that emphasizes using living organisms, substances from living organisms, or the body’s immune system against tumors, in an immunosuppressive tumor microenvironment. There are different treatment modalities of immunotherapy that are being exploited to be used alone or in combination with other conventional cancer therapies such as chemotherapy and radiation therapy for the purpose of increasing tumor reduction, minimizing the chances of relapse, and prolonging the longevity of a patient’s life [39]. A recent breakthrough in cancer research immunotherapy has been reported by scientists based on a clinical trial in which all patients under trial showed complete remission from colon cancer. This study was conducted on a small group consisting of 12 participants, all of whom had the same cancer mutation called mismatch repair-deficient colorectal cancer. This mutation is associated with 5–10 percent of colorectal cancer patients, per study. Cancer tumors in these patients had responded poorly to conventional chemo and radiation therapy. Dostarlimab is a monoclonal antibody that targets programmed cell death protein (PD-1) on the surface of T cells, increasing their sensitivity to the recognition and destruction of cancer cells. Cancer cells, in response, produce such molecules that bind and block PD-1 in order to hide from immune invasion. Dostarlimab acted by helping the immune system recognize cancer cells, decreasing the potential of cancer cells to evade the immune response [40].

## 2. Immunotherapeutic Approaches for the Treatment of Cervical Cancer Include

Immune checkpoint inhibitors.
Adoptive cell therapies.
Oncolytic virus therapy.
Cancer vaccines.

### 2.1. Immune Checkpoint Inhibitors

**Figure 2:**
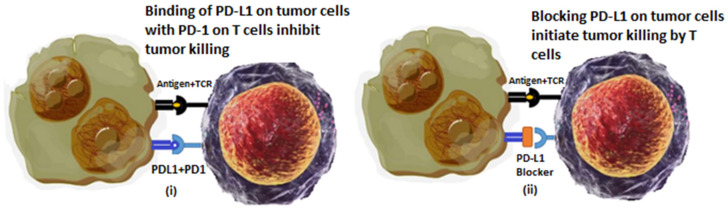
Immune checkpoint blockade therapy for suppression of cervical cancer. (**i**) Depicts immune evasion due to expression to immune checkpoint molecules. (**ii**) Shows action of immune checkpoint blocking molecule (such as pembrolizumab) that promotes T cell recognition and killing of tumor cells.

The discovery and therapeutic outcomes of immune checkpoint inhibitors are a turning point in the cancer treatment regimen. Despite undergoing a second line of chemotherapy or chemoradiation therapy, there was no guarantee of disease-free progression or an increase in the rate of survival for patients. Immune checkpoints are specific proteins expressed by some types of immune cells, particularly T cells. They promote self-tolerance and prevent indiscriminate immune actions in the body [41]. Cancer cells express these checkpoint molecules on a considerable fraction of tumors. According to a recent study, these checkpoints not only help cancer cells in immune evasion but also promote malignant behavior such as epithelial to mesenchyme transition, self-renewal, drug resistance, metastasis, enhanced energy metabolism, angiogenesis, and antiapoptotic behavior [42]. Cancer cells extensively express an immune checkpoint PD-L1 (programmed cell death protein-1) on their surface. The addition of inhibitors to PD-1 has dramatically enhanced disease-free progression and survival rates in cervical cancer patients [43]. A study was reported on a stage IV cervical cancer patient; this 38-year-old patient had progressed from the initial stage IB2 to stage 4 in just a few months. Following diagnosis, the patient received six cycles of cisplatin along with radiation therapy and brachytherapy for the initial diagnosis of cervical carcinoma. After lung metastasis, she was treated for stage 4 cervical cancer, but she was unable to tolerate bevacizumab or cisplatin due to renal issues and poor bone marrow reserves. Her tumor was diagnosed to be 100% programmed cell death ligand-1 (PD-L1) positive so she was treated with an FDA-approved pembrolizumab (FDA-approved for combination therapy in cervical cancer), which is a humanized monoclonal antibody that binds and blocks PD-1 (Figure 2) [44]. Following this treatment, the patient not only recovered but is reported to be disease free for more than 2 years without any problematic side effects [45]. Another immune checkpoint is CTLA4, also called CD152, which acts as an immune checkpoint and suppresses immune responses. Recent research comprising of ICI and radiotherapy in combination reported significant increase in therapeutic outcomes. They also disprove the common conception that radiation therapy suppresses the immune response and immunotherapy outcomes in cancer. According to preclinical research outcomes from a study that consisted of 101 patients, 70 patients received treatment with both ipilimumab and radiotherapy, while others received only ipilimumab. The results showed that, as compared to patients who received only ICI therapy, those who received synergistic treatment of ipilimumab and radiotherapy showed a better response to treatment and an increased overall survival rate [46]. This combination of ICI-like PD-1/PD-L1 with radiation has been reported to enhance local and distant efficacy. Moreover, the combination of radiation therapy with CTLA-4 and PD-1 stimulate the non-redundant immune response in cancer patients [47]. According to another study, the combination immunotherapy consisting of a low dose of nanomicelle-loaded paclitaxel and anti-PD-1 increased the infiltration of DCs and T cells at the tumor site and showed immune-dependent control of the tumor in cervical cancer patients [48]. Significant clinical activity in cervical cancer patients was reported when patients were treated with the combination of a PD-L1- and CTLA4-blocking moAb nivolumab and ipilimumab, respectively. This study named as CheckMate-358 exhibited promising clinical outcomes in cervical cancer patients with recurrent or metastatic cancer, regardless of PD-L1 status of expression [49]. Table 1 shows a few immune checkpoint inhibitors that have been explored for the treatment of cervical cancer.

### 2.2. Adoptive Cell Therapies (ACTs)

Adoptive T-cell therapy involves harvesting highly potent tumor-reactive T cells from patients and then expanding them in a laboratory setting to receive large amounts of patient-specific T cells, which can efficiently recognize and target tumor [52]. Until now, the three most accepted T cell therapies have included chimeric antigen receptor T cells (CAR-T), T-cell receptor-engineered T cells (TCR-T), and tumor-infiltrating lymphocytes (TILs) cell therapy. Among these ACT therapies, TIL has shown promising results in the treatment of metastatic cervical cancer because it is the least manageable and has minimal safety risks. Although this approach faces complications regarding its efficacy and specificity, in clinical practice many promising outcomes and achievements have been produced by adoptive T cell therapy. No adoptive cell therapy has been approved for solid tumors yet, but most attention has been paid to treating melanomas among solid cancers using ACT [7]. Unlike CAR-T and TCR-T cells, which can target a small number of antigens, TILs can target diverse phenotypes as they are made naturally from the TME of cancer. Natural T cell receptors (TCRs) are expressed on the surface of TILs that enable them to recognize tumor antigens of a patient in an MHC-dependent manner [53].

The first positive reported clinical trial of TILs in cervical tumors was reported in 2015 by Rosenberg et al. Their trial consisted of nine refractory metastatic cervical cancer patients receiving TIL therapy; these patients were not showing any improvement despite prolonged chemotherapy treatments. Out of these nine patients, one patient depicted partial remission, whereas two patients exhibited complete remission of the disease. The efficacy of this TIL-based therapy model for cervical cancer patients was 33.3%, emphasizing further improvements in the treatment regimen [54]. In phase II of a one-armed clinical trial by the same group in 2017, the efficacy of TILs was reported to be 27.8% (NCT01585428). In this trial, 18 HPV-positive cervical cancer patients received TILs therapy, out of which two patients showed complete remission, whereas three patients showed partial remission of disease [55]. In 20218 a lead researcher from the American cancer institute reported the complete remission of cervical cancer in two patients treated with TILs; they had a survival period of above 5 years. Another phase II clinical trial of of TILs in cervical cancer reported an objective effective rate of 28% (NCT01585428) [56]. Table 2 shows adoptive cell therapies based clinical trials in cervical cancer.

### 2.3. Oncolytic Viruses (OVs)

**Figure 3:**
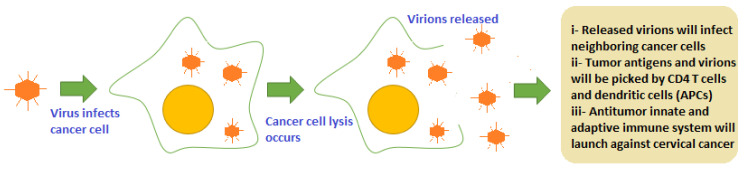
Oncolytic virus infection-mediated oncolysis of cervical cancer cells that leads to priming of both innate and adaptive immunity initiating a potent and viable immune response in tumor.

Viruses are obligate parasites and use host machinery for replication and survival. They have the ability to bind with specific receptors and are then internalized by endocytosis. Oncolytic viruses are a group of viruses that can target and bind to specific receptors, which are overly expressed on cancer cells (CD144, CD46, and CD44) [57]. Cancer cells have weak or deficient interferon (IFN) pathways, which make them more susceptible to infection with oncolytic virus as compared to normal cells. Viruses manipulate this defect of cancer cells and continue to divide in cells leading to lysis of tumor cells. Cellular lysis is mediated by CD8+ cytotoxic T lymphocytes, which recognize viral peptide epitopes presented by MHC-I molecules on infected cell surface receptors. The immune response is activated and lysis is also enhanced due to virions progeny released in the tumor microenvironment (picked by CD4 T cells and dendritic cells) and due to apoptosis activation (Figure 3) [58]. Until now, only two OVs have been approved for cancer treatment: so far, T-VEC (in USA) and Onyx-015 (in China) for metastatic melanoma and head and neck squamous carcinoma (HNSCC), respectively [59]. Table 3 shows the major oncolytic viruses being explored for the treatment of cervical cancer.

### 2.4. Cancer Vaccines

Therapeutic vaccines are another strategy to fight cervical cancer. They work by boosting the immune system against cancer. Live vector-based vaccines hijack the translational machinery of cancer cells and express specific antigens on tumor cells that elicit an immune response against cells [65]. TA-HPV (human antigen–human papillomavirus) is a vaccinia virus-based live vector vaccine. TA-HPV encodes mutated E6 and E7 onco-proteins of HPV16 and HPV18. Clinical studies have demonstrated the therapeutic efficacy of TA-HPV in inducing a potent HPV-specific cytotoxic T-cell reaction in cervical cancer patients [66].

Live vector-based vaccine options also include several bacterial vector-based immunogens such as several HPV therapeutic vaccine candidates that have bacterial vector bases, including *Listeria monocytogenes*, *Lactobacillus casei*, *Lactobacillus plantarum* and *Lactococcus lactis* [67]. Listeria has provided promising results as it can infect macrophages and release a pore-forming toxin listeriolysin O (LLO), which helps it to complement mediated immune clearance, giving it a chance to replicate in cytoplasm. Its antigen peptides are expressed on the cell surface via MHC-I and MHC-II receptors, leading to the activation of both helper T cells and cytotoxic T cells [68].

A dendritic cell (DCs)-based vaccine, which is a type of whole cell-based vaccine, works by presenting HPV antigens to immune players of both innate and adaptive immunity. DCs are loaded with HPV antigens and these preloaded DCs are then delivered to patients [69]. Along with HPV antigens they can be loaded with siRNAs too in order to evade apoptosis and maximize the life of DCs [70]. A phase I clinical trial was conducted in stage I1 and IIa cervical cancer patients who were treated with a DC-based vaccine. These patients were subcutaneously injected with a DC vaccine carrying keyhole limpet hemocyanin (KLH) and full length HPV16/18 E7, which led to CD4+ and a specific humoral immune response against cervical cancer [71].

The tissue antigen-cervical intraepithelial neoplasia vaccine (TA-CIN) is a protein-based vaccine that constitutes a single fusion protein to elicit a cell-based immune response against L2 proteins and HPV16 E6, and E7 antigens. Several early phase clinical trials have proved the immunogenicity and safety of this vaccine in cervical cancer patients. The efficacy of TA-CIN is being investigated in cervical dysplasia, including both high- and low-grade squamous intraepithelial lesion [72]. Due to its safety profile, TA-CIN is being investigated in combination with anti-PD-1 therapy in a clinical trial (NCT05132803) for recurrent HPV16-associated cervical cancer [73].

## 3. Discussion

Immunotherapy holds promising therapeutic outcomes as it comes with increased tumor suppression combined with decreased toxic side effects as compared to conventional therapies. Immune checkpoint blockade therapy is now being implicated as the first-line treatment in metastatic cancer, and extensive Phase III clinical trials are being carried out to maximize the therapeutic outcomes of this treatment modality for locally advanced, metastatic cervical cancer and for other malignancies. Adoptive T-cell therapy or cell-based therapies have added the aspect of the personalized treatment of cancer in which different modes such as CAR-T, TCR-T, and TILs can be used as a single treatment option or in combination to eliminate cervical cancer completely at early stages. Monoclonal antibodies include a broad range of therapeutic options due to their ability to target multiple receptors involved in tumor progression, angiogenesis, and metastasis. They are ideal candidates to be used in synergy with other conventional treatment options such as ipilimumab, and in combination with radiotherapy is proving to be a significant anticancer treatment in clinical trials. Oncolytic viruses have gained much attention due to their ability of modification to target and kill cancer cells only. They are being exploited to be used in synergy with chemotherapeutic agents and immune modulators for broad spectrum efficacious outcomes against cervical cancer and other malignancies. Cancer vaccines have gained popularity as they come with the least side effects, comparatively, and have both preventive and therapeutic implications in cancer treatment regimen. Further research on these immunotherapy approaches and combination therapies can help to target cancer stem cells, which are major contributors to cancer relapse, eliminating cancer completely with prolonged disease-free survival and enhanced quality of life post cancer treatment.

## 4. Conclusions

The revolutionizing immunotherapy modality has given mankind hope for the complete eradication of cervical cancer from the world. Immune system perturbations play a pivotal role in cervical cancer progression, metastasis, and relapse. A deeper understanding of the cross talk between immune players and the tumor microenvironment can pave the way to achieving disease-free survival for cervical cancer patients. Immunotherapy reprograms the immune system into the better detection and neutralization of tumor cells. It comes with a plethora of strategies such as immune checkpoint blockade, monoclonal Abs, oncolytic viruses, cell-based therapies, and cancer vaccines to train the immune system into better surveillance and eradication of cancer cells. Immunotherapy drugs such as pembrolizumab have been approved by the FDA for use in combination with chemotherapy for the treatment of cervical carcinoma. Many other immunotherapy options are giving significant therapeutic outcomes when used alone or in combination; this gives hope that cancer may soon become a thing of past.

## Figures and Tables

**Table 1:** Immune checkpoint inhibitors for the treatment of cervical cancer.

Target	Agent	Advantages	Limitations
**CTLA-4**	Ipilimumab	Used in solid and hematological malignancies Produces durable response even in advanced stage cancers Least side effects and patient compliance as compared to chemotherapy Have Biomarkers available to predict therapy response [50]	Therapeutic efficacy restricted to a limited number of patients and specific cancer in some cases Less effective in cancer with “cold” TME. Autoimmune-like toxicities: Nephritis Cytopenias Fatigue Myocarditis Hepatitis Hypophysitis Hypothyroidism Nephritis Uveitis Pneumonitis [51]
**PD-1**	Cemiplimab		
	Pembrolizumab		
	Nivolumab		
**PD-L1**	Durvalumab		
	Atezolizumab		
	Avelumab		

**Table 2:** Shows 5 registered trials of TILs on the web of clinical trials.

No.	NCT Number	Title	Phases	Enrollment	Combined Treatment	Status
1	NCT01585428	Immunotherapy using tumor-infiltrating lymphocytes for patients with metastatic human papillomavirus-associated cancers	Phase 2	29	-	Completed
2	NCT03108495	Study of LN-145, autologous tumor-infiltrating lymphocytes in the treatment of patients with cervical carcinoma	Phase 2	138	Pembrolizumab	Recruiting
3	NCT04674488	TILs for treatment of metastatic or recurrent cervical cancer	Phase 1	15	-	Recruiting
4	NCT05107739	Study of DeTIL-0255 in adults with advanced malignancies	Phase 1		-	Recruiting
5	NCT04443296	Study of tumor-infiltrating lymphocytes following CCRT in the treatment of patients with cervical carcinoma	Phase 1	10	CCRT Concurrent chemoradiotherapy	Active, not recruiting

**Table 3:** Oncolytic viruses being exploited for treatment of cervical cancer.

Virus	Description	Mechanism	Clinical Condition	Reference
**Adenovirus**	Non-enveloped virus with 90–100 nm size, have icosahedral nucleocapsid, which contain double-stranded DNA genome	Target tumor antigens specifically. Different Ad virus species bind with different receptors including CAR, αvβ5 integrin, HSPG, VCAM-1, MHC-Iα2	ONYX-015 (FDA-approved in China) is used in synergy with standard chemotherapy agents 5-fluorouracil and cisplatin to treat head and neck squamous carcinoma. Another virus similar to Onyx-015 (E1B-55K/E3B-deleted), H101 is tested promising for use in combination with radiation therapy to treat metastatic cervical cancer.	[60] [61]
**Newcastle Disease virus**	Single-stranded, negative sense, enveloped RNA virus. Causes contagious bird disease	Targeted replication in interferon-defective cancer cells by binding with Sia receptors on tumor cells. Avoids problem of pre-existing immunity	NDV selectively kills cervical cancer cells by inducing ROS-mediated apoptosis. NDV triggers both innate and adaptive immune response in cervical cancer TME by causing inflammation and recruitment of CD4+ and CD8+ immune responses.	[62]
**Vaccinia virus**	Linear double-stranded DNA genome containing enveloped virus. Approx 360 × 270 × 250 nm in size	Can squeeze through leaky tumor vascular for targeted infection tumor cells. Binds with MARCO receptor (macrophage receptor with collagenous structure)	It has been reported that oncoVV (vaccinia varus) encoding AVL *Aphrocallistes vastus* lectin (AVL) genes enhanced the cytotoxic effect of oncolytic vaccinia virus (oncoVV) in cervical cancer both in vitro and in vivo.	[63]
**Herpes simplex virus(HSV)**	Linear, double stranded DNA genome virus with approximately 152 kbp length	Binds with at least 3 receptors which are over expressed/abnormally expressed on cancer cells. Receptors are HVEM, nectin-1 and 3-OS-HS.	As reported, triple-mutated, third-generation HSV therapy was targeted for HPV16- or HPV18-associated cervical carcinoma in which human Hela xenograft and TC-1 syngeneic models were studied. It was found that oncolytic HSV greatly inhibited cervical tumor growth, mediated apoptosis, and turned cervical cancer tumor “hot” for immune targeting.	[64]
